# NF-kB mediated down-regulation of collagen synthesis upon HEMA (2-hydroxyethyl methacrylate) treatment of primary human gingival fibroblast/*Streptococcus mutans* co-cultured cells

**DOI:** 10.1007/s00784-014-1304-4

**Published:** 2014-09-09

**Authors:** R. Grande, S. Pacella, M. Di Giulio, M. Rapino, V. Di Valerio, L. Cellini, A. Cataldi

**Affiliations:** 1 Dipartimento di Farmacia, Università G. d’Annunzio, Chieti-Pescara, 66100 Chieti, Italy; 2 Dipartimento di Medicina e Scienze dell’Invecchiamento, Università G. d’Annunzio, Chieti-Pescara, 66100, Chieti, Italy; 3 Istituto di Genetica Molecolare del CNR, Unità di Chieti, Chieti, Italy

**Keywords:** NF-kB, Type I collagen, HEMA, HGF/*Streptococcus mutans* co-cultured cells

## Abstract

**Purpose:**

In vitro studies have evidenced the cytotoxic effect of HEMA (2-hydroxyethyl methacrylate), the most common component of dental resin-based restorative material, which is released within the oral cavity, on eukaryotic cells such as gingival fibroblast and epithelial cells. However, since the presence of microorganisms within the oral cavity cannot be excluded and little is known about the interactions occurring between eukaryotic cells and the human oral microbiota, our attention has been addressed to investigate the effect of 3 mM HEMA on the molecular mechanisms driving the response of human gingival fibroblasts (HGFs) co-cultured with *Streptococcus mutans*.

**Methodology:**

HGF/*S. mutans* co-culture has been set up in our lab, and upon HEMA treatment, *S.mutans* and HGF cells’ viability and adhesion along with type I collagen gene and pro-collagen I, Bax, Bcl2, nuclear factor kB (NF-kB), IkBα, pIkBα protein expression by PCR, Western blotting and ELISA assays have been investigated.

**Results:**

HEMA treatment determines a significant decrease of type I collagen protein production, even in the presence of *S. mutans,* in parallel to a decrease of cell viability and adhesion, which seem to be regulated by NF-kB activation. In fact, when SN50, NF-kB-specific pharmacological inhibitor, is added to the culture, cell proliferation along with collagen synthesis is restored.

**Conclusion:**

The modulation exerted by *S. mutans* on the cytotoxic effect of HEMA suggests that within the oral cavity, the eukaryotic/prokaryotic cell interactions, maintaining the balance of the environment, allow HEMA to perform its adhesive and bonding function and that the use of a co-culture system, which simulates the oral cavity organization, improves the knowledge concerning the biocompatibility of this dental material.

## Introduction

Among the components of dental resin materials, which are released into aqueous or organic solvents, 2-hydroxyethyl methacrylate (HEMA) is included [1, 2]. This wetting monomer agent, which facilitates penetration of the hydrophilic component, like BisGMA and UDMA into hydrophilic environments like dentin [3], in vivo can be released into the oral cavity, as observed in dental practice [4, 5], or into the pulp space [6] inducing toxic effect on eukaryotic cells, such as gingival fibroblast and epithelial cells [7–15].

Although the in vitro response to HEMA evidences immunogenic, teratogenic, genotoxic, inflammatory, oxidative cell damage and apoptosis in the eukaryotic cells of the oral cavity [4, 16], it should not be forgotten that also numerous bacterial strains with commensal and pathogen significance are represented in this environment [17–19] and such material might affect interactions between eukaryotic and prokaryotic cells.

Oral streptococci encompass friends and foe bacteria. Imbalance in the indigenous microbial environment generates oral diseases, and under proper conditions, commensal streptococci can switch to opportunistic pathogens that initiate disease and damage to the host. *Streptococcus mutans* is one of the strains naturally present in the human oral microbiota and is the most important bacterium related to the formation of the dental caries, thanks to its capability to produce an acidic environment and to form sticky biofilm [20–23]. However, some authors have not detected this species in children patients with caries and when found, it was part of a complex microbial community [24, 25]. Dental caries process is associated to the development of dental plaque or biofilm in which the bacterial community, embedded in the extracellular polysaccharide (EPS) matrix adhered to the tooth surface, causes the demineralization of dental hard tissue through the production of acids from dietary sugars. The presence of a cariogenic species, like *S. mutans,* and/or biomaterials, like HEMA, alone and in combination with each other, might induce a cellular response of the oral tissues, in terms of gene and protein expression [26, 27].

Nuclear factor kB (NF-kB) is a dimeric transcriptional factor which activates a great variety of genes involved in stress response, immune cell activation, cancer occurrence, cell growth, inflammation, programmed cell death and survival [28–36]. NF-kB activation is regulated by a multi-subunit IkB kinase (IKK-B) complex which phosphorylates IkBα marking it for degradation by ubiquitin pathway, so that NF-kB dimer can translocate to the nucleus, bind DNA and activate transcription genes [37]. Even though NF-kB is also involved in the protection against apoptosis induced by HEMA in mouse embryonic fibroblasts [38] and in dental pulp stromal cells and oral keratinocytes [39, 40], no evidence concerning the role played by NF-kB intracellular signalling pathway in the response to HEMA in the presence of bacterial strains is reported.

Thus, since in our lab a eukaryotic/prokaryotic co-culture model has been set up [41, 42], the effect of 3 mM HEMA on the molecular mechanisms driving the response of human gingival fibroblast (HGF) co-cultured with *S. mutans* has been evaluated. We have chosen to use 3 mM HEMA according to previous studies by Falconi et al. [43], in which 3 mM HEMA has been demonstrated to be responsible for a reduction of cell viability lower than 50 %, and by Kurata [16], which shows a drastic anti-proliferative effect of 1 mM HEMA followed by a further increase at 3 and 5 mM in fibroblasts derived from human pulp.

In addition, being fibroblast cells producer of collagen type I, which allows cell-cell or cell-substrate adhesion [28] and nuclear transcription factor NF-kB a regulator of collagen biosynthesis [29, 30], here we report the response disclosed by HGF cells to HEMA in terms of adhesion and proliferation in the presence of *S. mutans*.

## Materials and methods

### Bacterial strain and growth condition

The reference strain *S. mutans* ATCC 25175 has been cultured in Trypticase soy broth (Oxoid, Milan, Italy) at 37 °C for 18–24 h under anaerobic atmosphere. The overnight culture has been diluted 1:10 (*v*/*v*) in Dulbecco’s modified Eagle’s medium (DMEM, Euroclone, Milan, Italy) antibiotic and serum-free plus 1 % (*w*/*v*) sucrose and refreshed for 2 h at 37 °C in an orbital shaker (Julabo SW-20 C, Milan, Italy) at 160 rpm in aerobic condition. The broth culture has been adjusted to 0.5 McFarland, corresponding to approximately 1.5 × 10^8^ CFU/ml and used for co-culture set-up.

### Culture of human gingival fibroblasts

HGFs have been obtained from fragments of healthy marginal gingival tissue taken from the retromolar area during surgical extraction of impacted third molars. Signed informed consent has been obtained from the donors. Tissue fragments have been immediately placed in Dulbecco’s modified Eagle’s medium (DMEM)/F12 for at least 1 h, rinsed three times in phosphate-buffered saline solution (PBS), minced into small tissue pieces and cultured in DMEM/F12, 10 % foetal bovine serum (FBS), 1 % penicillin and streptomycin and 1 % fungizone. Cells have been maintained at 37 °C in a humidified atmosphere of 5 % (*v*/*v*) CO_2_ and after 4–8 passages have been seeded into 96-well and 6-well culture plates (Nunc, EuroCloneSpA, Life-Sciences-Division, Milan, Italy) with DMEM containing 10 % FBS, penicillin and streptomycin. The NF-kB/IkB pharmacological inhibitor SN50 (50 μM) (Enzo Life Sciences, Lausen, CH4415 Switzerland) [44] has been added to the cells 45 min before *S.mutans* and HEMA.

### HEMA treatment and co-colture set-up

2-Hydroxyethyl-methacrylate (HEMA) (Sigma-Aldrich, Milan, Italy) stock solution 1 M in ethanol has been filtered through 0.2-μm pore size filters and diluted in DMEM to obtain a medium containing 3 mM HEMA according to Falconi et al. [43]. The ethanol concentration in the medium used for the experiments was lower than 0.3 %. The co-culture assays,have been performed in 25-cm^2^ flasks for the adhesion assay and 75 and 150 cm^2^ for multiplex reverse transcription-PCR (MRT-PCR) and protein extraction, respectively. The HGFs have been seeded in culture plates in DMEM containing 10 % FBS, 1 % penicillin and streptomycin in a humidified atmosphere of 5 % (*v*/*v*) CO_2_ at 37 °C. When cells have reached confluence, the medium has been removed and cell washed with PBS. The bacterial cultures, standardized in DMEM 1 % sucrose, have then been added to HGF confluent cells together with DMEM containing 3 mM HEMA. Just the DMEM has been added to the control cultures. Moreover, *S. mutans* ATCC 25175 and HGFs have been assayed alone in DMEM 1 % sucrose with and without HEMA. The flasks have been incubated for 24 h in humidified atmosphere of 5 % (*v*/*v*) CO_2_ at 37 °C. The choice of a single incubation time point is associated to a cell viability reduction of *S. mutans* evaluated by live/dead staining and subsequent microscopic observation after 24 h of incubation.

In conclusion, for the evaluation of the effect of HEMA and *S. mutans* ATCC25175 alone and in combination with each other on HGF cells, trypan blue dye exclusion test has been performed in the following experimental conditions:

- HGF (control)
- HGF plus HEMA
- HGF plus *S. mutans* ATCC 25175
- HGF plus HEMA plus *S. mutans* ATCC 25175

For each assay, the experimental design has been carried out at least for two independent experiments and each experiment performed in triplicate.

### LDH cytotoxicity assay

HGFs have been seeded at 200,000 cells/well on 24-well plates. After 24 h, culture medium has been replaced with 1 ml of DMEM 1 % sucrose containing standardized bacterial cultures (final volume 1 ml). After 30 min at 37 °C, medium has been harvested and lactate dehydrogenase-based assay (LDH assay, TOX-7, Sigma-Aldrich, St. Louis, MO) has been performed on culture media according to manufacturer’s instructions. As positive control, cells have been lysed with Triton 1 %. Each test has been performed in quadruplicate.

Assessment of cytotoxicity has been calculated according to the formula:

$$ \%\;\mathrm{LDH}\;\mathrm{released}\kern0.5em =\kern0.5em \left[\left(\mathrm{A}-\mathrm{B}\right)/\left(\mathrm{C}-\mathrm{B}\right)\right]\kern0.5em \times 100, $$

with A = LDH activity of sample, B = LDH activity of untreated cells and C = LDH activity of the positive control.

### *S. mutans* cell viability evaluation and adhesion assay

The effect of 3 mM HEMA on streptococcal adhesion on HGFs has been evaluated by colony forming unit (CFU) counts. *S.mutans*, grown as mentioned before, has been co-coltured with HGFs with and without 3 mM HEMA in 25-cm^2^ cell culture flasks. After 24 h of incubation, the planktonic bacteria have been removed while sessile cells, adhered to HGFs, have been rinsed with PBS and removed by using a cell scraper plus 1 mL sterile PBS. The obtained sample has been homogenized at 10,500 rpm for 30 s, at 25,000 rpm for 40 s and 30,000 rpm for 40 s, by using a homogenizer (*IKA*®*T10 basic Ultra*, Staufen, Germany) for separating bacteria aggregates. The number of bacteria has been determined by dilution plating on Trypticase soy agar (Oxoid, Milan, Italy). The plates have been incubated for 24 h in anaerobic atmosphere and in aerobic atmosphere for another 24 h at 37 °C. Each assay has been performed for three independent experiments in duplicate.

### RT-PCR assay

For the evaluation of gene expression and cell viability, HGFs have been rinsed with PBS, trypsinized and processed for trypan blue dye exclusion test, which selectively identifies dead fibroblasts in blue, and counted in a Burker chamber after 24 h of incubation.

With regard to RNA isolation, total RNA has been extracted from HGF cells by using QIAzolLysis Reagent [45] and DNase I digestion has been performed according to the manufacturer’s instructions (Sigma-Aldrich, Milan, Italy). Total RNA (2 μg) has been electrophoresed on a 1 % formaldehyde agarose gel, to evaluate RNA integrity. For the evaluation of type I collagen gene expression, a multiplex reverse transcription-PCR (MRT-PCR) with the housekeeping gene glyceraldehyde 3-phosphate dehydrogenase (GAPDH) has been carried out. The oligonucleotide primers used are as follows: ha (1)-coll. 1-f (5′-CTGACCTTCCTGCGCCTGATGTCC-3′); ha(1)-coll 1-r (5′-GTCTGGGGCACCAACGTCCAAGGG-3′) for collagen-1(36); and hGAPDH-f (5′-CAACTACATGGTTTACATGTTC-3′) and hGAPDH-r (5′-GCCAGTGGACTCCACGAC-3′) for the housekeeping gene GAPDH [46]. MRT-PCR has been performed by using the One-Step RT-PCR Kit (Qiagen, Milan, Italy) in a final reaction volume of 25 μl containing 160 ng of total RNA, 1× Qiagen buffer, 500 mM of each dNTP, 0.6 mM of each primer and 5 U of One-Step RT enzyme. RT-PCR Master Mix has been performed in a 2700 thermocycler (Applied Biosystems, Foster City, CA, USA) for 30 min at 50 °C and 15 min at 95 °C for RT and initial PCR activation, respectively. The amplification of Collagen I/GAPDH gene consisted of 15 min at 95 °C and then 35 cycles of 30 s at 94 °C, 1 min at 63 °C, 1 min at 72 °C, with a final 10 min extension at 72 °C. To assess the specificity of the primers used in the experiment, a sample containing just *S. mutans* ATCC 25175 RNA has been processed as negative control: no amplification products have been detected.

PCR products (6 μl) have been analysed by electrophoresis in a 2 % (*w*/*v*) agarose gel at 100 V for 45 min. Gels have been stained with ethidium bromide and photographed. The expected sizes of the amplification products have been approximately 300 bp for Collagen-I and 181 bp for GAPDH, respectively.

The relative level of expression for each gene has been calculated as the ratio between the signal obtained in the area of the target band and the signal of the GAPDH, by scanning densitometry by using the GELDOC XRS system by the QuantiOne 1-D analysis software (BIORAD, Richmond, CA, USA). Two independent experiments, performed in duplicate, have been carried out.

### Western blotting analysis

Total cell lysates (60 μg) have been electrophoresed on a 10 % sodium dodecyl sulphate (SDS)-polyacrylamide gel and transferred to nitrocellulose membrane. Nitrocellulose membranes, blocked in 5 % BSA, 10 mmol/L Tris-HCl pH 7.5, 100 mmol/L NaCl and 0.1 % Tween-20, have been probed with mouse anti β-actin and anti β-tubulin antibodies (Sigma, USA), goat pro-collagen I, mouse anti-BAX, mouse anti BCL2 antibodies (Santa Cruz, Santa Cruz Biotechnology, CA, USA), polyclonal anti NF-kB, IkBα and p-IkBα antibodies (Cell Signalling Technology) and then incubated in the presence of specific enzyme-conjugated IgG horseradish peroxidase. Immunoreactive bands have been detected by ECL detection system (Amersham Intl., Buckinghamshire, UK) and analysed by densitometry. Densitometric values, expressed as integrated optical intensity (IOI), have been estimated in a CHEMIDOC XRS system by the QuantiOne 1-D analysis software (BIORAD, Richmond, CA, USA). Obtained values have been normalized basing on densitometric levels of internal β tubulin and α-actin.

### ELISA assay

Type I collagen secretion has been evaluated on both experimental discs and polystyrene plates at 24 h of culture by means of ELISA assay (Human Collagen type I ELISA, COSMO BIO CO., LTD), according to manufacturer’s instructions.

### Statistical analysis

The significance of the differences recorded in the assay performed with and without HEMA in each experimental condition has been evaluated using Student’s *t* test. Probability levels of <0.05 have been considered statistically significant. Statistical analysis has been performed using the analysis of variance (ANOVA). Results have been expressed as mean ± SD. Values of *p* < 0.05 have been considered statistically significant.

## Results

**Fig. 1:**
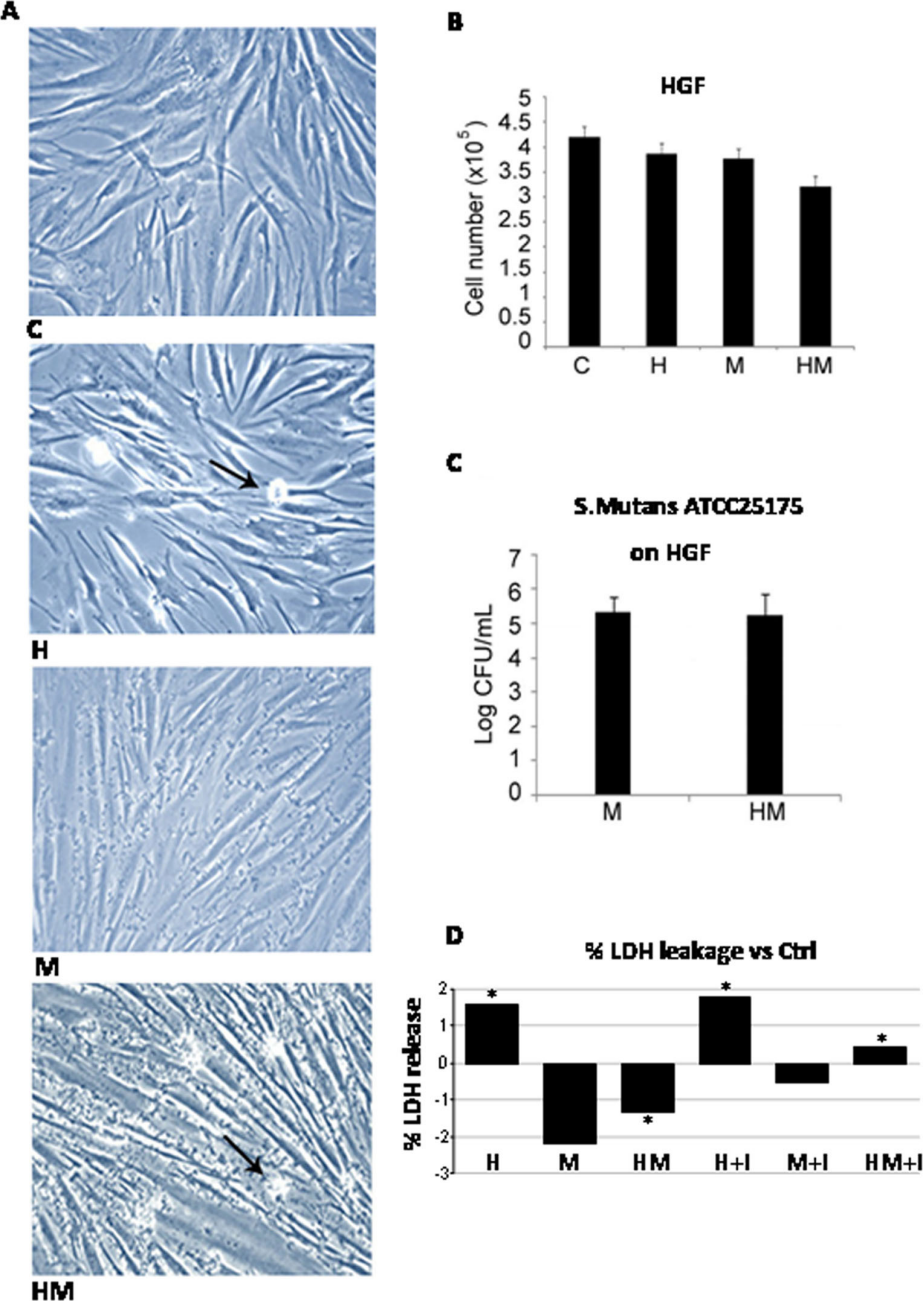
**a** Light phase contrast microscopy of co-cultured human gingival fibroblasts (HGF)/*S. mutans* ATCC 25175 in different experimental conditions. *Arrows* indicate dead cells. Magnification ×10. **b** Trypan blue dye exclusion test of primary cultures of HGF exposed for 24 h to 3 mM HEMA co-cultured or not in the presence of *S. mutans* ATCC 25175. Data are the mean of three separate experiments (±SD). **c** Effect of 3 mM HEMA on the viable growth of *S. mutans* ATCC 25175 in co-culture with human gingival fibroblast after 24 h of incubation. Viable growth of *S. mutans* ATCC 25175 adherent on HGF with and without HEMA. Data are the mean of three separate experiments (±SD). **d** LDH release in HGF in different experimental conditions. *Graph* represents the mean percentage ± SD of three experiments. *C* HGF (control), *H* HGF + HEMA, *M* HGF + *S. mutans* ATCC25175, *HM* HGF + HEMA + *S. mutans* ATCC 25175

**Fig. 2:**
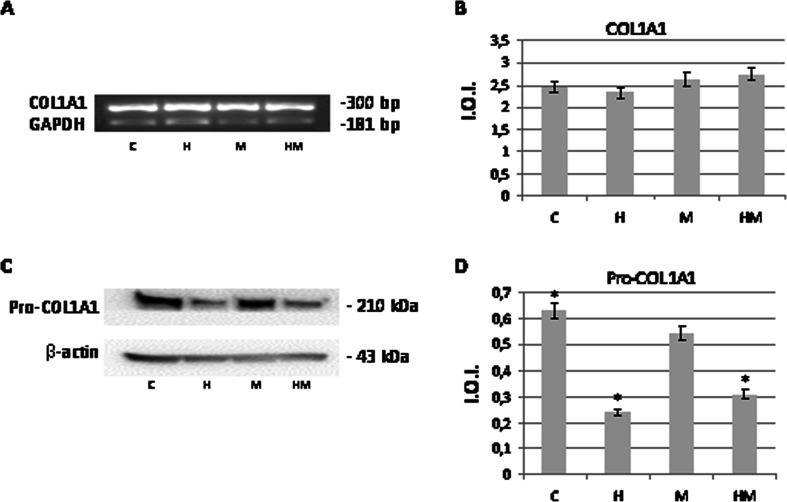
**a** Representative image of the expression of CoL1A1 normalized with the expression of the internal control GAPDH. **b** Densitometric analysis (±SD) of CoL1A1 gene expression. **c** Western blot analysis of procollagen I expression in HGF exposed for 24 h to 3 mM HEMA co-cultured or not in the presence of *S. mutans* ATCC 25175. Total protein (60 μg) has been loaded for each lane and the membrane probed with β-actin antibody to verify loading evenness. The blot is the most representative out of three independent experiments performed in duplicate. **d** Densitometric analysis (±SD) of Procollagen I protein expression. For legend, see Fig. 1; *H*, *HM* versus C pro-collagen I: *p* < 0.05

**Fig. 3:**
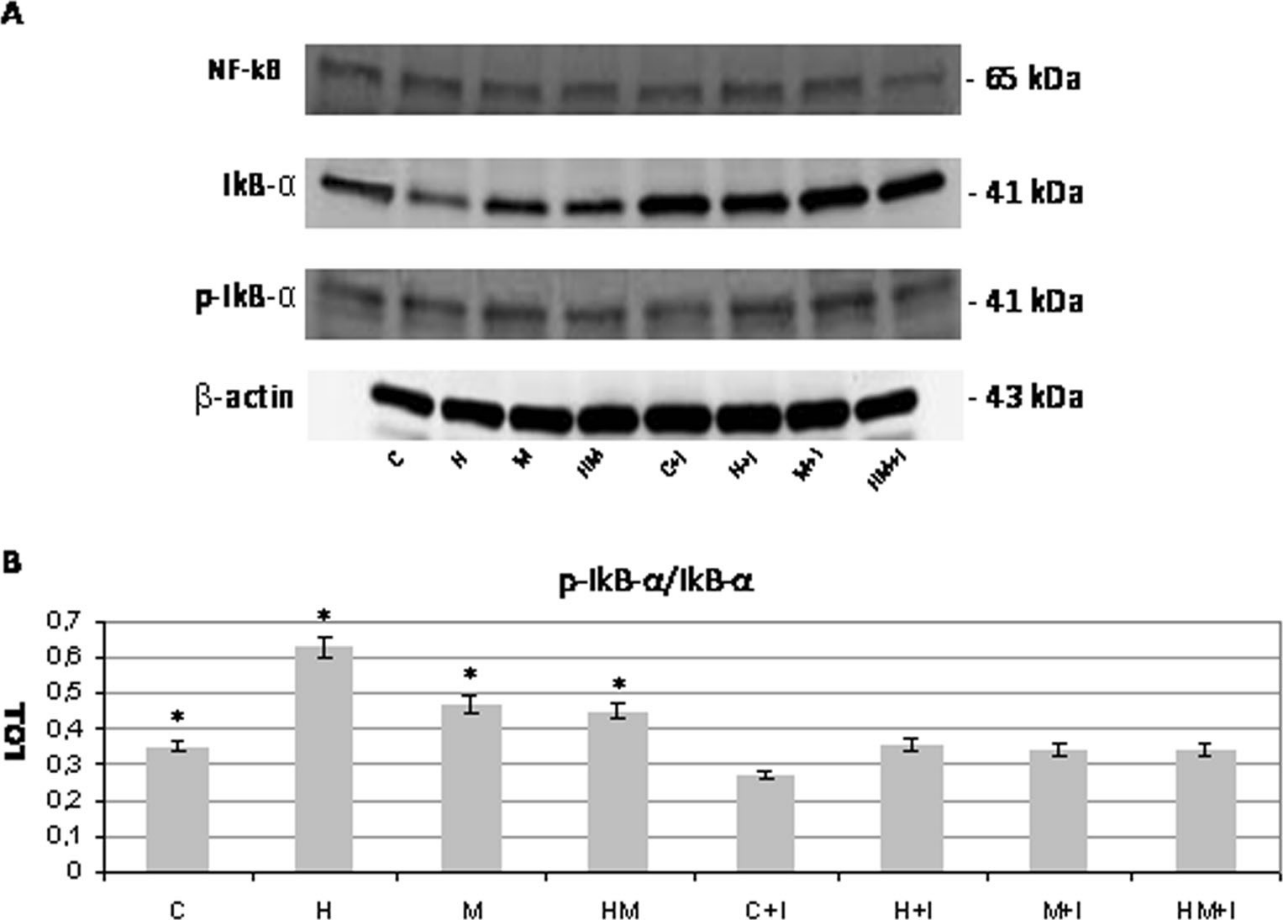
**a** Western blot analysis of NF-kB, IkBα and p-IkBα expression in HGF exposed for 24 h to 3 mM HEMA co-cultured or not in the presence of *S. mutans*. When required, the NF-kB/Ikb pharmacological inhibitor SN50 has been added to the culture 45 min before *S. mutans* and HEMA treatment. Total protein (60 μg) has been loaded for each lane and the membrane probed with β-actin antibody to verify loading evenness. The blot is the most representative out of three independent experiments performed in duplicate. **b** Densitometric analysis (±SD) of p-IkBα/IkBα ratio. For legend, see Fig. 1; *H*, *M*, *HM* versus *C* p-IkBα: *p* < 0.05

**Fig. 4:**
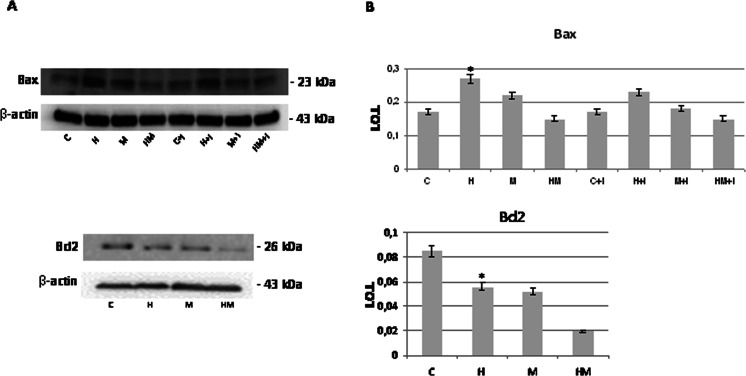
**a** Western blot analysis of Bax and Bcl-2 expression in HGF exposed for 24 h to 3 mM HEMA co-cultured or not in the presence of *S. mutans.* When required, the NF-kB/IkB pharmacological inhibitor SN50 has been added to the culture 45 min before *S. mutans* and HEMA treatment. Total protein (60 μg) has been loaded for each lane and the membrane probed with β-actin antibody to verify loading evenness. The blot is the most representative out of three independent experiments performed in duplicate. **b** Densitometric analysis (±SD) of Bax and Bcl-2 expression. For legend, see Fig. 1; *H*, *M*, *HM* versus *C* Bax: *p* < 0.05; *H*, *M*, *HM* versus *C* Bcl-2: *p* < 0.05

**Fig. 5:**
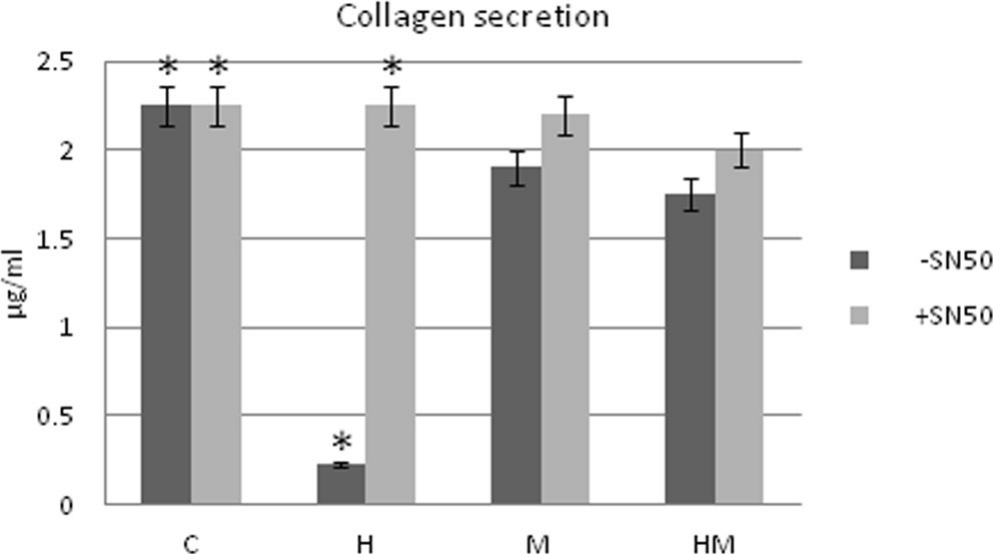
ELISA assay for type I collagen secretion of primary cultures of HGF exposed for 24 h to 3 mM HEMA co-cultured or not in the presence of *S.mutans*. When required, the NF-kB/IkB pharmacological inhibitor SN50 has been added to the culture 45 min before *S. mutans* and HEMA treatment. Secretion levels have been measured at 24 h and reported as micrograms per millilitre. The results are the mean (±SD) of three different experiments. H-SN50 versus C collagen secretion, *p* < 0.05; H + SN50 versus C collagen secretion, *p* < 0.05

Light phase contrast microscopy analysis evidences that fibroblasts loose adhesion upon HEMA treatment also in the presence of *S. mutans*, while trypan blue dye exclusion test shows that cell viability is still high after 24 h of treatment in all the experimental conditions (Fig. 1a, b). No effect of HEMA on bacteria viability, assayed by CFU counts, is observed, while when an LDH assay, which is considered a cytotoxicity assay, has been performed, the cytotoxic effect of HEMA is reduced in the presence of *S. mutans* (Fig. 1c, d). Even though no difference is evidenced when procollagen I gene expression is evaluated in HGF by using GAPDH as housekeeping gene (Fig. 2a, b), Western blotting analysis shows a significant decrease of procollagen I protein level in HGF samples treated with HEMA alone and in the presence of *S. mutans* for 24 h, while no difference is revealed between untreated cells and co-cultures without HEMA (Fig. 2c, d). Since a direct correlation has been demonstrated between collagen gene suppression and NF-kB activation, we have investigated if HEMA is able to activate this signalling pathway, which plays a key role in the regulation of stress response genes. Both NF-kB and IkB expressions have been tested revealing no differences in the different experimental conditions, while an increased p-IkBα/IkBα ratio is shown in HGF treated with HEMA and also in co-cultures in the presence or not of HEMA (Fig. 3). In the cells which loose adhesion apoptotic events seem to be switched on, as shown by increased level of pro-apoptotic Bax protein, as well as by decreased level of anti-apoptotic Bcl-2 protein, in those cells treated only with HEMA (Fig. 4), coming back to basal levels in the presence of *S. mutans*, even though when an Annexin/PI assay, which detects early apoptosis, has been performed, no difference has been evidenced in the different experimental conditions (data not shown). Interestingly, when, to add specificity to such results, the NF-kB/IkB pharmacological inhibitor SN50 is administered to the co-cultures 45 min before *S. mutans* and HEMA treatment, collagen secretion, evidenced by ELISA assay, is restored (Fig. 5) as well as LDH activity and p-IkBα/IkBα ratio are decreased (Figs. 1 and 3). Moreover, Bax expression does not undergo modification in the presence of SN50 inhibitor (Fig. 4).

## Discussion

Oral and systemic diseases are frequently related to the effects of released dental restorative materials and of adherence of microbial species to oral cavity tissues. Even though a lot of studies concerning the effects exerted by HEMA, TEGDMA and other dental biomaterials on oral tissue are published [1, 2, 4], it is of great interest to know how the presence of pathogenic bacterial community on oral tissue may interfere with eukaryotic gene expression and protein synthesis in the response to biomaterials. As Johansson A. et al. [47] suggest, the oral disease may not be due to the presence of oral pathogen and/or toxic substances, but it is fundamental to evaluate the interactions between biomaterials/bacteria/host.

Moreover, since the interaction between microbial and oral tissue is almost unexplored, in this study, we have tested the effects of HEMA monomer on HGF/*S. mutans* co-cultured cells.

Human gingival fibroblasts are connective cells which can either mediate the adhesion to bone structure by producing procollagen I protein or interact with microbial cells. Thus, with the aim to investigate the human gingival fibroblasts’ response both to HEMA and bacteria in terms of adhesion and viability, in our lab, an HGF/*S. mutans* co-culture model has been set up. In particular, while in the presence of HEMA viability is not significantly modified in each experimental condition, adhesion, shown by procollagen I expression, is significantly reduced in the presence of HEMA plus or minus *S. mutans*, not affected by *S. mutans* alone. Even bacteria viability, assayed by CFU counts, does not show any modification upon HEMA treatment. Since collagen synthesis inhibition has been elsewhere reported to be regulated by NF-kB activation [30], here we report increased p-IkBα/IkBα ratio in parallel to reduced collagen biosynthesis, which justifies reduced cell adhesion, in response to HEMA, while when HGFs are incubated with *S. mutans* or HEMA and *S. mutans*, this effect is less evidenced. The reduced cell adhesion can justify the increased Bax expression level in parallel to Bcl2 decrease, less evident in the other experimental conditions, even though apoptotic events do not seem to occur in this experimental model. When the NF-kB/IkB pharmacological inhibitor SN50 is added to the culture, collagen secretion is restored in all the experimental conditions, as revealed by ELISA assay, as well as LDH activity, p-IkBα/IkBα ratio is decreased upon HEMA treatment suggesting a key role for NF-kB in the anti-proliferative response of human gingival fibroblasts. Moreover, upon inhibitor treatment, no modification is observed in Bax expression suggesting that cells do not undergo apoptosis.

Even though to improve this co-culture model in order to resemble the oral cavity environment, saliva should be added, as already reported by our group in the presence of *Streptococcus mitis* strains [48, 49], here it has not been included since it does not significantly modify the *S. mutans* adhesion capability in the presence of biomaterials, as elsewhere already described [50].

The evidence that HEMA reduces cell adhesion and viability of HGF cells and that this response is modulated by *S. mutans* presence and driven by the NF-kB signalling pathway suggests that in the oral cavity, the eukaryotic/prokaryotic cells’ interaction, maintaining the balance within the environment, allows HEMA to exert its adhesive and bonding function and that the use of a co-culture system, which simulates oral cavity organization, improves the knowledge concerning biocompatibility of this dental material.
